# The Ocular Manifestations of COVID-19 Through Conjunctivitis

**DOI:** 10.7759/cureus.12218

**Published:** 2020-12-22

**Authors:** Abhimanyu S Ahuja, Bryan A Farford, Madeline Forouhi, Rama Abdin, Manisha Salinas

**Affiliations:** 1 Medicine, Florida Atlantic University Charles E. Schmidt College of Medicine, Boca Raton, USA; 2 Family Medicine, Mayo Clinic, Jacksonville, USA; 3 Research, Mayo Clinic, Jacksonville, USA

**Keywords:** covid-19, protective eyewear, ocular health

## Abstract

There is growing evidence that the novel coronavirus (SARS-CoV-2) is capable of transmission through the eye. Research suggests that infection by SARS-CoV-2 can produce an inflammation of the conjunctiva, which leads to redness and itchiness of the eyes. Furthermore, viral particles have been detected in conjunctival secretions of SARS-CoV-2 patients who present with conjunctivitis and is likely another mode of transmission. A 53-year-old male presented with a complaint of left eye irritation and upper eyelid swelling for the past 24 hours. The right eye had mild irritation but no lid swelling. The left upper eyelid was erythematous, swollen and had crusting along the lashes. There were mild inflammation and injection of the conjunctiva. The initial diagnosis was blepharitis, and it was recommended that he continue with the warm compresses, and doxycycline 100 mg to use if the symptoms worsened or did not improve. The patient underwent SARS-CoV-2 PCR testing as a requirement for travel the next day and was found to be positive for the virus. Over the following days, he developed fatigue and rhinitis but clinically improved within six days of his initial presentation. Physicians and health care workers should be aware of the ocular manifestations of SARS-CoV-2 to make a timely diagnosis of infected individuals. While requirements vary across institutions, it is highly recommended that healthcare workers consistently wear appropriate eye protection when interacting with patients to reduce the spread of disease and potential impact on ocular health from SARS-CoV-2. Additionally, to prevent ocular transmission, all healthcare workers should be immediately educated on the importance of eye protection.

## Introduction

The novel coronavirus, SARS-CoV-2, has pervaded the global population since its initial outbreak in Wuhan, China and, in less than a year, has developed into a global pandemic. One of the first Chinese physicians to call attention to the peculiar uptick in pneumonia cases in Wuhan in late December 2019 was an ophthalmologist, who was reported to have contracted the virus from an asymptomatic glaucoma patient he treated in his clinic. Due to the unprecedented nature of the virus, a worldwide quest to uncover the cellular and subcellular mechanisms behind transmission of the virus has elucidated some general detail regarding routes of ophthalmic transmission of SARS-CoV-2 but leaves much to be explored. Given the evidence of ophthalmic transmission of previous coronaviruses, we posit that the lack of thorough understanding of viral transmission mechanisms in the eye-specifically the conjunctiva-has resulted in an underestimation of the risk of ophthalmic viral spread. In this analysis, we present the ophthalmic mechanisms of viral transmission including in previous coronavirus strains, the current clinical ophthalmic manifestations of SARS-CoV-2, considerations for personal protective equipment (PPE) use, as well as speculation on the significance of viral transmission via the eye.

Only a handful of studies have explored possible ophthalmic routes of SARS-CoV-2 transmission as great concern surrounds the potential for the virus to be transmitted via the eye. As previously mentioned, existing hypothesized mechanisms of SARS-CoV-2 include ophthalmic modes of transmission such as through the exposed conjunctiva and generated aerosols, via fomites and subsequent contact with one’s own exposed mucosa, and through ocular fluids, such as tears [1,2]. Clinically, viral conjunctivitis has been shown to occur in previous coronavirus strains, including the initial severe acute respiratory syndrome coronavirus (SARS-CoV) [3]. Research has shown that SARS-CoV-2 maintains the potential to be transmitted via aerosols (particles suspended in a gas, such as the air) which can make contact with the conjunctiva [3]. Viral particles have also been detected in conjunctival secretions of SARS-CoV-2 patients who present with conjunctivitis. Thus, it has been surmised that SARS-CoV-2, like many upper respiratory viruses, has the potential ability to transmit through the mucosal membranes of the eye [4]. This notion especially highlights the issue of viral dissemination to the conjunctiva itself (via aerosolized particles and fomites) as well as the presence of viral DNA fragments in ocular fluids such as tears, both of which do not escape our notice as strikingly threatening routes of transmission. As we await further data which expands on ophthalmic viral transmissibility about distance and air circulation, a continued cautious approach in settings such as examination rooms is essential. Additionally, in the last few months, a debate has taken place over the controversy as to whether SARS-CoV-2 is found in tears. As more data emerge, however, evidence suggests that SARS-CoV-2 has been detected in tears of individuals infected with the virus, which is consistent with previous similar viruses such as SARS-CoV RNA [3,5].

Ophthalmic clinical presentation of COVID-19 can inform both our understanding of SARS-CoV-2 mechanism of transmission as well as indications of virulence which impact guidelines established for preventative measures in both private and public environments. The most prevalent symptoms of COVID-19 seen clinically include fever, cough, sore throat, and fatigue [6]. However, the ophthalmic manifestations of the virus should not be overlooked. A study analyzing the symptoms and progression of COVID-19 in 38 hospitalized patients in Yichang Central People’s Hospital found that 12 of the 38 patients had ophthalmic manifestations of conjunctivitis; for example, irritation of the eyes, epiphora, and increased ocular secretions [7]. These symptoms were most prevalent in patients with more severe disease patterns [7]. Notably, one of the patients within the study group presented with conjunctivitis as the first symptom of the virus [7].

Since SARS-CoV-2 is transmitted by contact with infected droplets, the use of proper PPE can help reduce the transmission of the virus [8]. It is recommended that all healthcare employees that will have direct patient contact wear eye protection in addition to an appropriate mask. Suitable eye protection includes full-length face shield, safety goggles or safety glasses. Individuals that require glasses for vision should wear appropriate eye protection over their glasses. For reusable protective eyewear, the eyewear should be cleaned and disinfected with hospital-grade disinfectant after doffing [9].

## Case presentation

**Figure 1:**
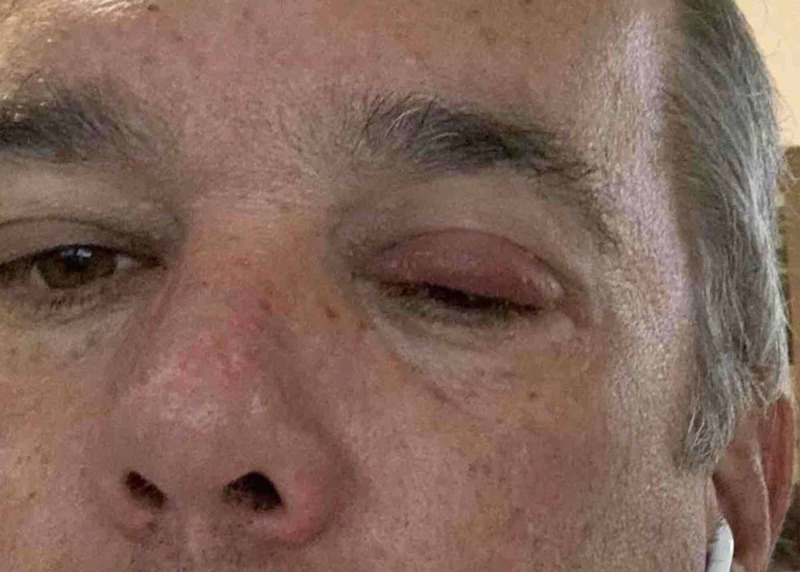
Patient Presenting with Eyelid Swelling

A 53-year-old male, with no known medical history, presented with a complaint of left eye irritation and upper lid swelling for the preceding 24 hours. The right eye had mild irritation, but no lid swelling (Figure 1). Patient photograph displaying mild inflammation and infection of the conjunctiva]. The patient denied having any associated symptoms, including vision changes, fever, rhinitis, cough, fatigue or sinus congestion. At home, he was using warm compresses to assist with the symptoms, and this provided temporary relief, but it did not reduce or resolve the overall symptoms. On examination, the left upper eyelid was erythematous, swollen and had crusting along the lashes. There were mild inflammation and injection of the conjunctiva. The right eyelid was normal, and there was no noticeable discharge. The right conjunctiva had faint erythema but was otherwise normal.

The initial diagnosis was blepharitis, and it was recommended that he continue with the warm compresses. Furthermore, he was given a prescription for doxycycline 100 mg to use if the symptoms worsened or did not improve. The following day the patient underwent SARS-CoV-2 PCR testing as a requirement for travel and was found to be positive for the virus. He subsequently contacted the clinic with an update on his diagnosis. Over the following days, he developed fatigue and rhinitis but clinically improved within six days of his initial presentation.

## Discussion

**Figure 2:**
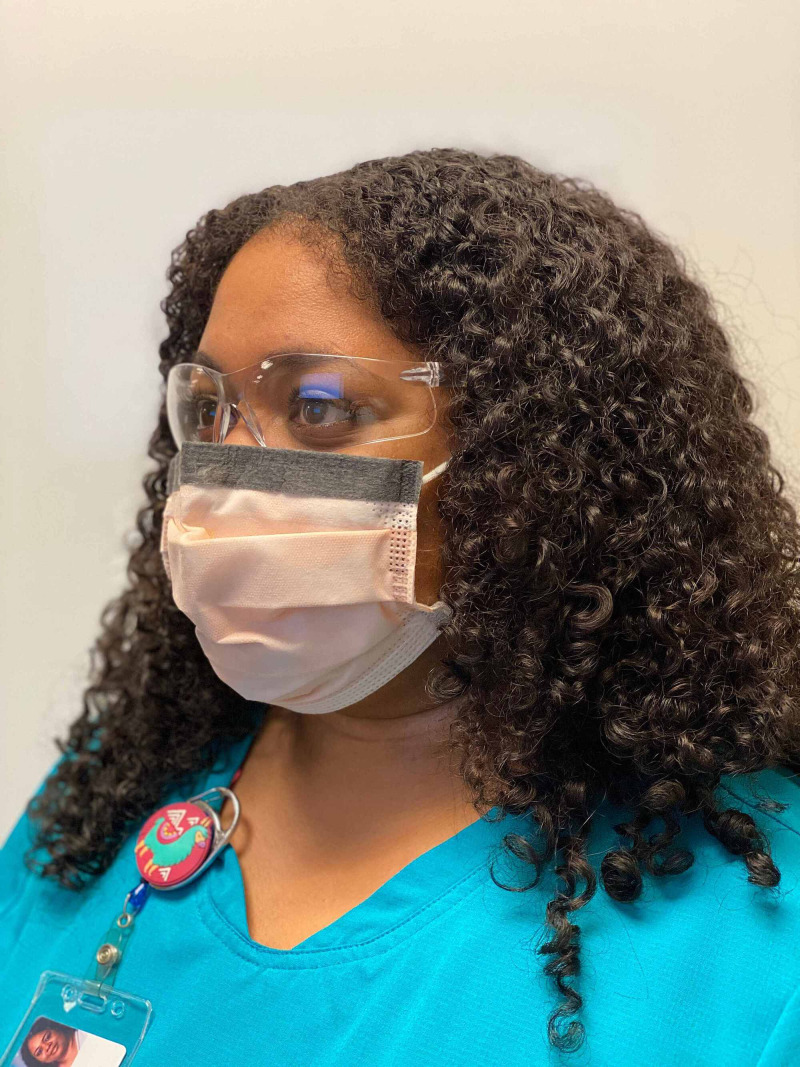
Safety Glasses

**Figure 3:**
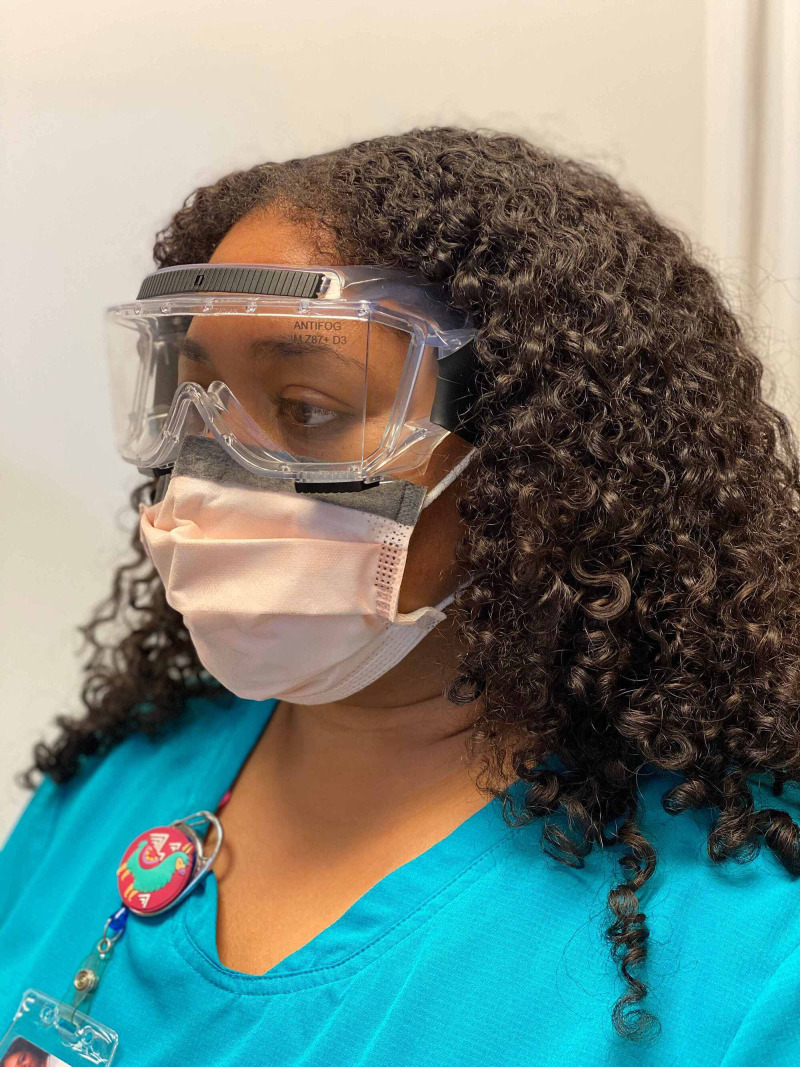
Safety Goggles

**Figure 4:**
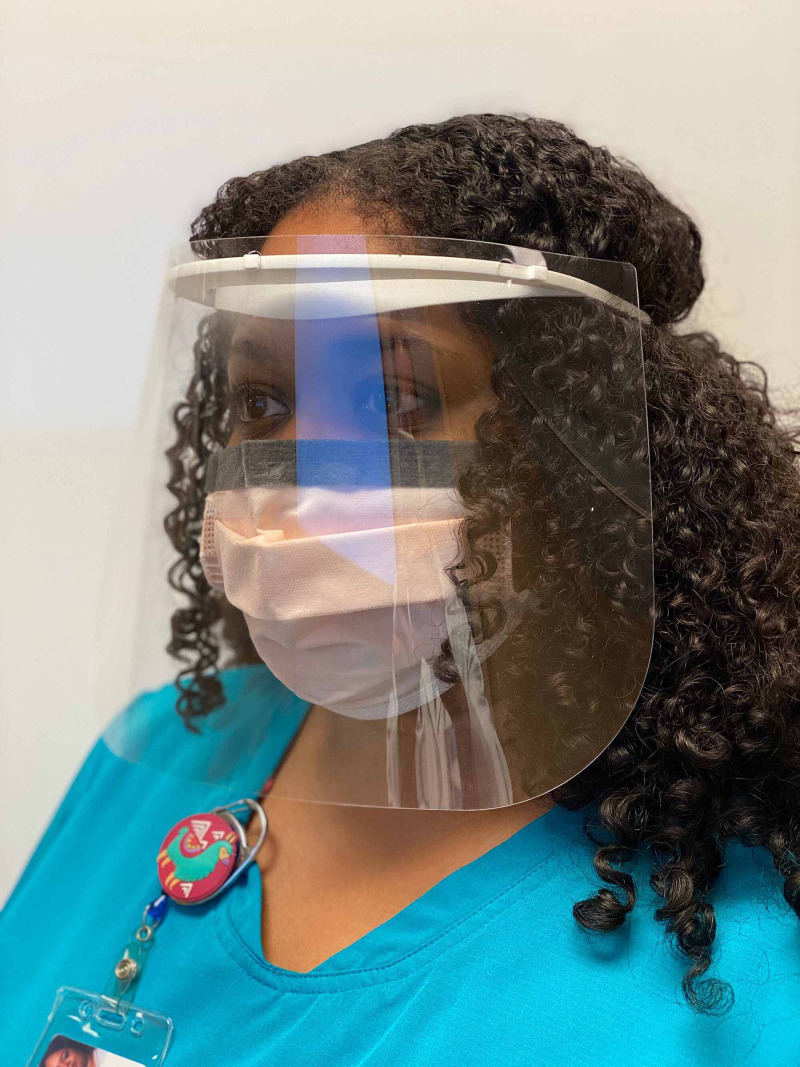
Full Face Shield

Though much is left to be determined regarding the novel coronavirus--especially its ophthalmic implications--the tremendous efforts of researchers in the last several months have provided us with some level of clarification as to the manifestation of the disease in the eyes and the level of precaution we must take in order to prevent the further transmission of the virus. Evidence of ophthalmic transmission of past coronavirus strains implores an investigation into the ability (virulence) of and mechanisms by which the novel coronavirus to spread through the eye. In addition to the presentation of conjunctivitis in several COVID-19 patients, the presence of viral particles on surfaces and within respiratory droplets, other aerosols, and ocular secretions poses a much more threatening means of ophthalmic transmission of SARS-CoV-2 than has previously been considered. Additional exploration may better and more specifically inform preventative practices. Nonetheless, health care workers should be aware of the appropriate usage of PPE for optimal prevention of disease transmission, particularly as it relates to ocular health and infection] [10]. Figures 2, 3, 4 illustrate appropriate donning of eye protection.

This case report builds on previous work that has found infection by SARS-CoV-2 can produce an inflammation of the conjunctiva, which leads to redness and itchiness of the eyes. Conjunctivitis appears to be the most prevalent ophthalmic manifestation of the disease and could potentially be one of the first symptoms of the virus. However, it has been demonstrated that patients infected with SARS-CoV-2 have presented with signs of blepharitis including lid margin hyperemia and/or telangiectasia, crusted eyelashes, and meibomian orifices alterations, potentially owing to the ability SARS-CoV-2 to alter the microenvironment and destabilize the tear film on the ocular surface epithelial cells and glands [11]. Without further research on the ocular symptoms related to SARS-CoV-2, it is possible that the virus could be overlooked in its early stages and the symptoms may be misidentified as more common causes of conjunctivitis (such as an allergic reaction or bacterial origin) rather than manifestations of the infection.

Not all patients presenting with conjunctivitis, but without the common symptoms of COVID- 19, will be examined by a medical professional since conjunctivitis is not usually sight-threatening. Therefore many people choose to forego a doctor’s appointment and self-treat the symptoms instead. As a result, people who may be experiencing conjunctivitis as an initial symptom of the novel coronavirus could be left undiagnosed and may continue to transmit the virus unknowingly. This is compounded by the fact that the pandemic has affected the possible examination processes, as in-person examinations have been limited, potentially making it more challenging to make a differential diagnosis.

Data regarding the number of patients who, after experiencing initial symptoms of conjunctivitis, developed the more commonly known symptoms of COVID-19 is scarce [12]. Thus, it is imperative to recognize the less commonly identified symptoms of the virus, such as ocular symptoms, as they could prove to be early indicators of the disease. As precautionary guidelines associated with COVID-19 begin to relax, it is necessary to continue to study the ophthalmic manifestations of SARS-CoV-2 as it appears general practitioners and ophthalmologist maybe some of the first physicians to treat early symptoms of the virus [12]. Importantly, to prevent infection in an in-person healthcare setting, the practice of correct utilization of PPE is necessary for both the patient and the physician. PPE, including protective eyewear, is vital for physicians and health care workers that are working in close proximity to patients.

## Conclusions

As much of the initial evidence is anecdotal, many questions remain regarding the relationship between SARS-CoV-2, clinical ophthalmic manifestations of the virus, and quantitative detail regarding the ophthalmic transmission of the virus. Further studies into the conjunctival mechanisms of SARS-COV-2 transmission and detection of the virus in tissues or fluids of the eye would prove valuable in tailoring precautionary strategies to prevent further spread of the virus. Until more evidence is available, however, all healthcare workers should don protective eyewear during direct patient care.
